# Protective efficacy of a recombinant Newcastle disease virus expressing glycoprotein of vesicular stomatitis virus in mice

**DOI:** 10.1186/s12985-016-0481-y

**Published:** 2016-02-24

**Authors:** Minmin Zhang, Jinying Ge, Xiaofang Li, Weiye Chen, Xijun Wang, Zhiyuan Wen, Zhigao Bu

**Affiliations:** State Key Laboratory of Veterinary Biotechnology, Harbin Veterinary Research Institute, Chinese Academy of Agricultural Sciences, 427 Maduan Street, Harbin, 150001 People’s Republic of China

**Keywords:** Vesicular stomatitis virus, Glycoprotein, Recombinant Newcastle disease virus, Vaccine

## Abstract

**Background:**

Vesicular stomatitis virus (VSV) causes severe losses to the animal husbandry industry. In this study, a recombinant Newcastle disease virus (NDV) expressing the glycoprotein (G) of VSV (rL-VSV-G) was constructed and its pathogenicity and immune protective efficacy in mouse were evaluated.

**Results:**

In pathogenicity evaluation test, the analysis of the viral distribution in mouse organs and body weight change showed that rL-VSV-G was safe in mice. In immune protection assay, the recombinant rL-VSV-G triggered a high titer of neutralizing antibodies against VSV. After challenge, the wild-type (wt) VSV viral load in mouse organs was lower in rL-VSV-G group than that in rLaSota groups. wt VSV was not detected in the blood, liver, or kidneys of mice, whereas it was found in these tissues in control groups. The mice body weight had no significant change after challenge in the rL-VSV-G group. Additionally, suckling mice produced from female mice immunized with rL-VSV-G were partially protected from wt VSV challenge.

**Conclusions:**

These results demonstrated that rL-VSV-G may be a suitable candidate vaccine against vesicular stomatitis (VS).

## Background

Vesicular stomatitis (VS) is caused by the vesicular stomatitis virus (VSV), which belongs to the genus *Vesiculovirus* of the family *Rhabdoviridae* and presents lesions in the mucous membranes of the mouth and nose and the epithelium of the feet and teats [1]. VSV has been detected in swine, cattle, horses, and other animals in the U.S. several decades ago [1]. In America, VSV has caused significant economic losses due to decreased milk and meat production, quarantines, trade barriers, and livestock market closures [2, 3]. This virus could spread between hoofed animals and rodents via insect vectors [4]. Vesicular stomatitis in humans is a uniformly non-fatal influenza-like illness [1].

Vaccination is preferentially used to prevent and control the disease in human and animals [5–9]. Inactivated VSV vaccines with aluminum hydroxide or oil as adjuvants have been tested in the United States of America and in Colombia according to the OIE Terrestrial Manual [10]. Additionally, a commercial bivalent inactivated VSV vaccine containing antigens against the New Jersey (NJ) and Indiana 1 (IND1) viruses were tested [11]. However, to induce high levels of neutralizing antibodies and protect animals from challenge by the virulent virus, the VSV antigens need to be concentrated by ultracentrifugation on sucrose gradient and then be inactivated [11], which is not convenient for commercial production because it increases costs and producing-process. Furthermore, immunization with inactivated vaccine is indistinguishable from natural VSV infection. The VSV Glycoprotein, which is the only protein on the viral envelope, plays crucial roles in attachment, fusion and entry into host cells [12]. G protein is highly immunogenic, and the target of neutralizing antibodies [13–15]. Immune responses induced by the expression of the VSV G subunit and DNA vaccines were tested in the laboratory, however, DNA vaccines couldn’t induce satisfactory neutralizing antibody titers [16], and subunit vaccines in general do not prime effectively for cell mediated immunity [15]. Live-vectored vaccines induce both humoral and cell-mediated immunity, which generally provide longer immune protection than inactivated or subunit vaccines [13, 14]. A recombinant vaccinia virus expressing VSV G protein provided partial protection against VSV challenge in cattle [15]. The Newcastle disease virus (NDV) genome is simple and easy to manipulate. It can be grown to high titers in chicken embryos for vaccine production. It has a strict host range and viral replication is restricted in mammals [17]. Its safety has been demonstrated in many animal models, including the African green monkey, rhesus monkey, pig, mouse, cattle, and chicken [18–26]. Its pre-existing immunity and maternal antibody against mammalian paramyxoviruses does not interfere with the replication of NDV, because it is antigenically distinct from the mammalian paramyxoviruses. NDV has been actively developed and used for the control of human and animal diseases in recent years [18–22, 25–31].

In this study, a recombinant NDV expressing the G protein of VSV was constructed. To the best of our knowledge, this is the first study on a NDV-based VSV vaccine. The pathogenicity and protective efficacy of this recombinant virus were analyzed and the results showed that the recombinant virus was safe in mice and could induce high titers of neutralizing antibody that protected adult or suckling mice from VSV challenge.

## Results

### Expression of VSV G protein by rL-VSV-G

**Fig. 1:**
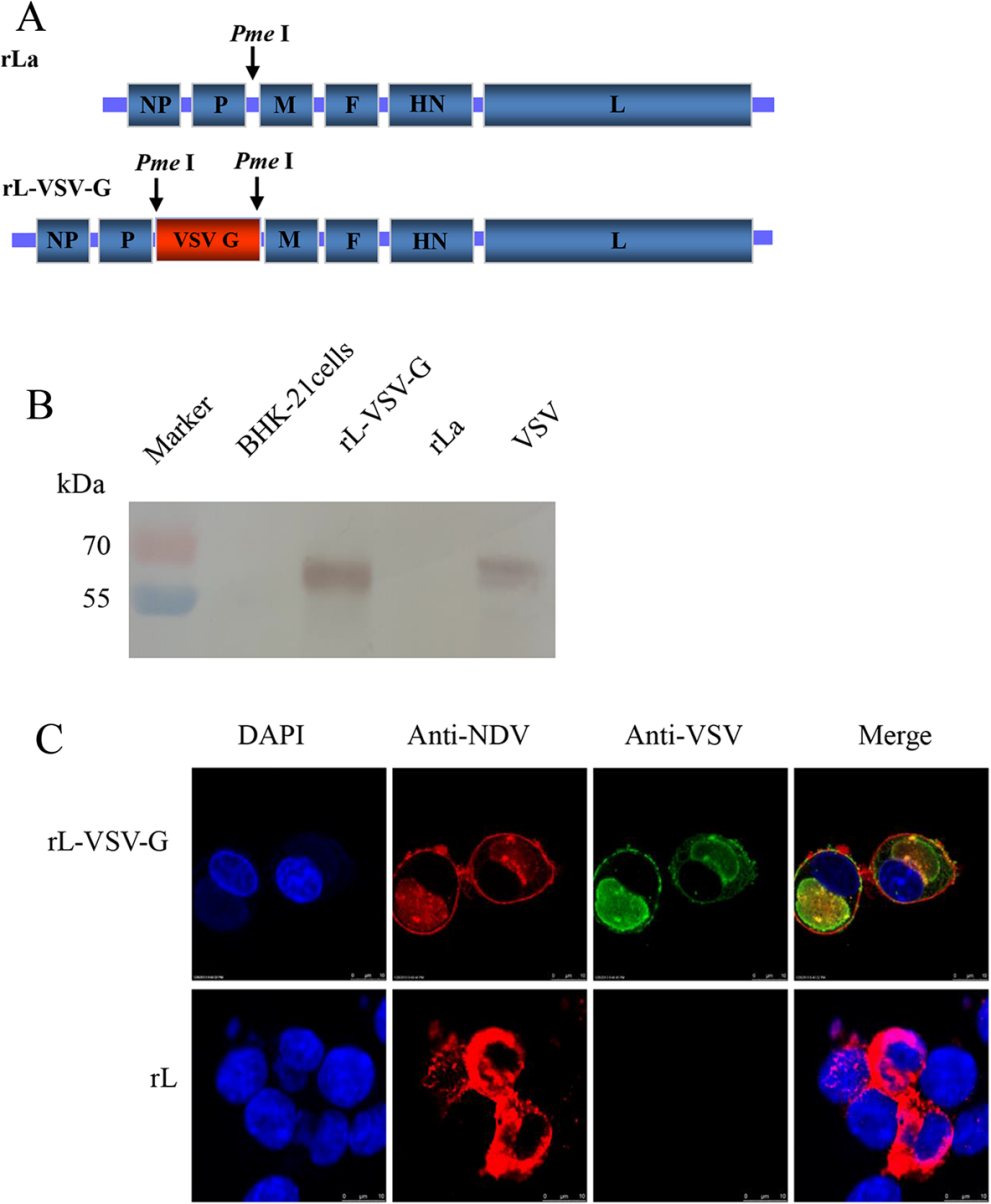
Construction and identification of rL-VSV-G. **a** Schematic representation of the rLaSota genome and VSV G inserted between the P and M genes. **b** Western blot assay of expression of VSV G. BHK-21 cells were infected with rLaSota or rL-VSV-G at a MOI = 1. After 24 h, cells were collected and lysed, the cell lysate was separated by SDS-PAGE and immunoblotted with mouse anti NDV antibodies or VSV G monoclonal antibody. **c** Immunofluorescence analysis of VSV G protein expression. BHK-21 cells were infected with rLaSota or rL-VSV-G at a MOI = 0.01. After 24 h, the cells were fixed and then stained with chicken anti-NDV antibody or VSV G monoclonal antibody followed by incubation with FITC-conjugated goat anti-mouse antibody or a TRITC-conjugated rabbit anti-chicken antibody

VSV Indiana strain G gene ORF was inserted between P and M gene of NDV genome (Fig. 1a). rL-VSV-G virus was recovered entirely from this cDNA using established reverse genetics procedures [22]. To confirm the expression of VSV G, BHK-21 cells were infected with rL-VSV-G at a MOI of 1. Cells infected with rL-VSV-G or rLaSota total proteins were detected by incubation with the monoclonal antibody against VSV G by Western blot. The Western blot assay demonstrated that rL-VSV-G reacted strongly with monoclonal antibodies against VSV G, producing a band of about 60 kDa, which is equal to the molecular mass of VSV G. However, the vector rLaSota did not react with the VSV G monoclonal antibodies and no band was detected (Fig. 1b). BHK-21 cells were also infected with rL-VSV-G at a MOI of 0.01, and at 48 h after infection, the cells were fixed and incubated with VSV G protein monoclonal antibody (Sigma, USA) or mouse anti-NDV antibody followed by staining with FITC-conjugated goat anti-mouse antibody or TRITC-conjugated rabbit anti-chicken antibody. Confocal immunofluorescence results confirmed the expression of VSV G protein in infected cells (Fig. 1c).

Furthermore, the genetic stability of VSV G gene in rL-VSV-G was assessed by 10 serial passage of the virus in SPF chicken eggs and confirmed by RT-PCR and immunofluorescence assay. The results demonstrated VSV G gene could be stably maintained and expressed (data not shown).

### Pathogenicity in poultry and mice

To determine the pathogenicity of rL-VSV-G in poultry, the mean death time (MDT), intracerebral pathogenicity index (ICPI), and intravenous pathogenicity index (IVPI) were determined in embryonated SPF chicken eggs or in SPF chickens according to the OIE Manual (OIE) [32]. The results indicated rL-VSV-G kept nonvirulent in poultry comparing with LaSota virus (data not shown).

The pathogenicity of rL-VSV-G was evaluated in mice that had been treated using two different delivery routes, intranasal instillation and intramuscular injection. The mice were inoculated with rL-VSV-G or LaSota at a dose of 10^7^ TCID_50_ in 100 μl PBS and equal volume PBS was intramuscularly injected or intranasally inoculated as mock infection. Supernatant from homogenized tissues was inoculated in BHK-21 cells, and viral load in these tissues was determined by IFA.

**Fig. 2:**
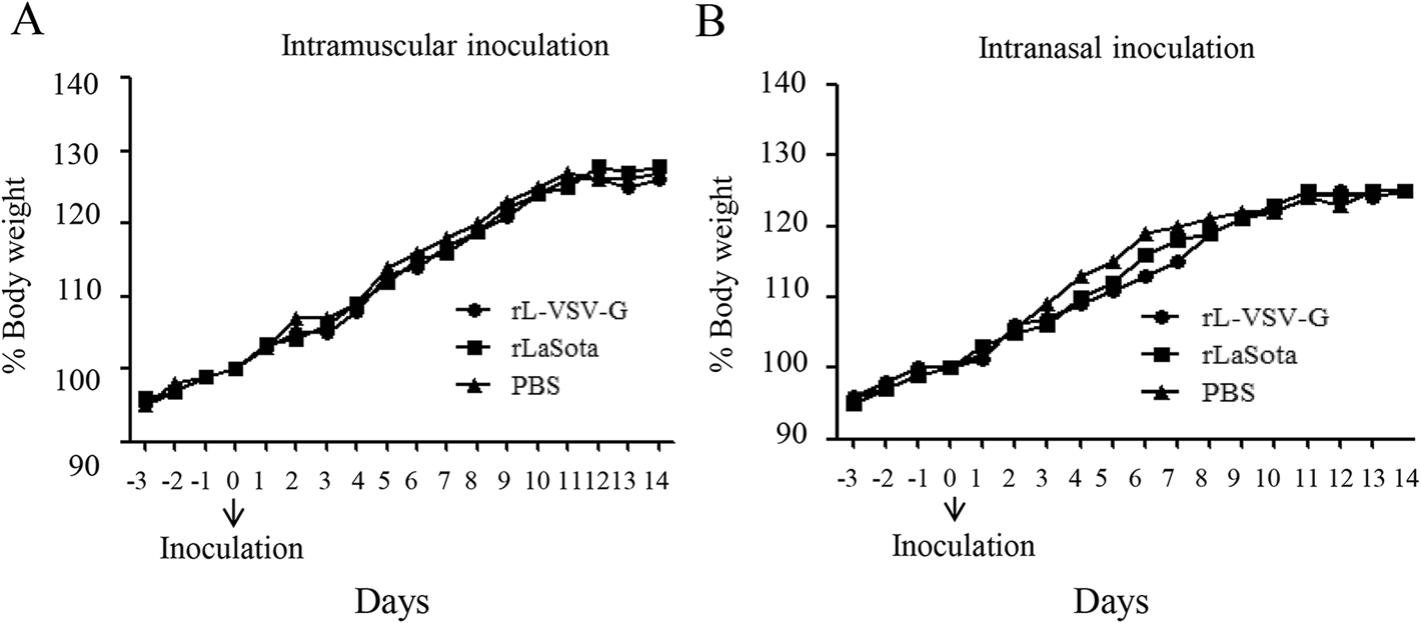
Changes in body weight in mice inoculated with rL-VSV-G. Mice were (**a**) intramuscularly injected or (**b**) intranasally inoculated with 10^7^ TCID_50_ of rL-VSV-G on day 0. Mice were observed and weighed daily from day −3 to day 14.. All mice survived the duration of the experiment. Body weight changes for each group are shown as ratios of the body weight at day 0, which was set as 100 %

The results show that all the mice inoculated with rL-VSV-G by either of two routes did not develop adverse reactions and and rL-VSV-G was not detected in the tissues of the brain, spleen, lung, liver, heart, or kidney (data not shown). There were no differences between rLaSota or rL-VSV-G infection groups with respect to changes in body weight after either intramuscular injection (Fig. 2a) or intranasal instillation (Fig. 2b). These data suggested that the VSV G inserting did not change the pathogenicity of vector virus NDV in mice and that rLaSota and rL-VSV-G had limited replication in major mice organs.

### rL-VSV-G-induced high-level VSV-specific antibodies and neutralizing antibodies in mice

**Fig. 3:**
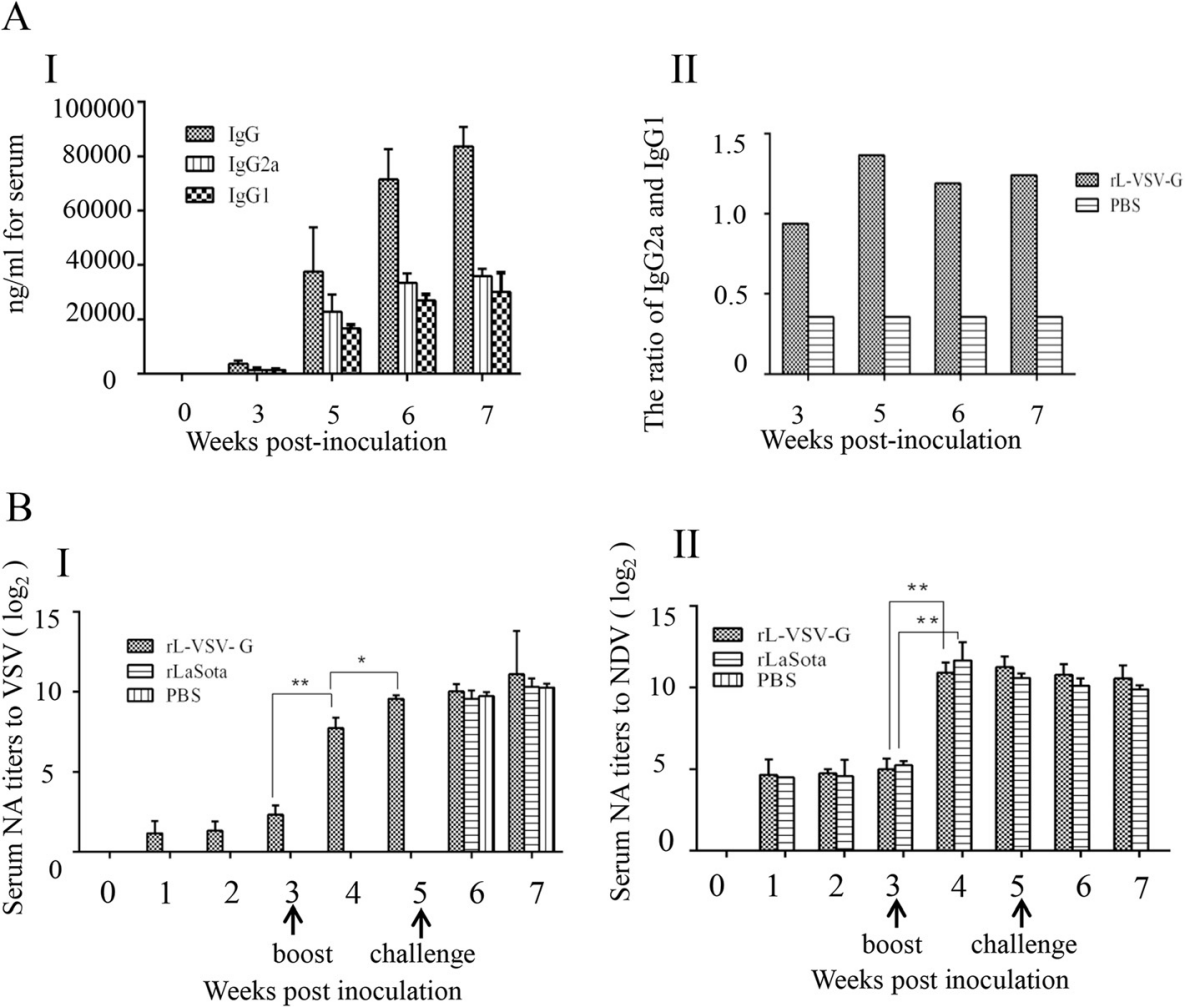
Serologic responses assays in mice. Serum Ig G antibody subtype and neutralization analyses. The antibody titers for each group are indicated as the means plus SD. (A-I) Quantity of IgG, IgG1, and IgG2a. (A-II) Ratio of IgG2a and IgG1. SN antibody titers for NDV and rL-VSV-G. The serum samples were collected at different times after vaccination and the (B-I) VSV and (B-II) NDV SN titers were measured. Statistically significant differences were determined using the t test. *, *P* < 0.05

Mice serum VSV-specific IgG, IgG1 and IgG2a were determined by ELISA after immunization. rL-VSV-G could induce specific IgG against VSV (Fig. 3A-I). At fifth week, rL-VSV-G group induced high level of IgG, IgG1, and IgG2a (Fig. 3A-I), which indicated skewing of systemic activity of T helper cell type 2 (Th2) and T helper cell type 1 (Th1) pathways, respectively. rL-VSV-G immunized mice tended to produce a Th1-type immune response (IgG2a/IgG1 > 1) (Fig. 3A-II), which may largely facilate the viral clearance in mice [33].

The serum neutralizing (SN) antibodies against VSV were detected in the rL-VSV-G group, and the titer was 1:2.25 at the first week after immunization (Fig. 3B-I). At the second week after booster immunization, the SN titers was 1:760 (Fig. 3B-I) in the rL-VSV-G group. SN antibody titers induced by rL-VSV-G showed no dramatic increases after challenge with wt VSV. This indicated that the wt VSV replication could be limited in rL-VSV-G immunized mice. The NDV SN titers were similar for both the rL-VSV-G and rLaSota groups (Fig. 3B-II).

### Assessment of protective efficacy of rL-VSV-G against wt VSV challenge

**Fig. 4:**
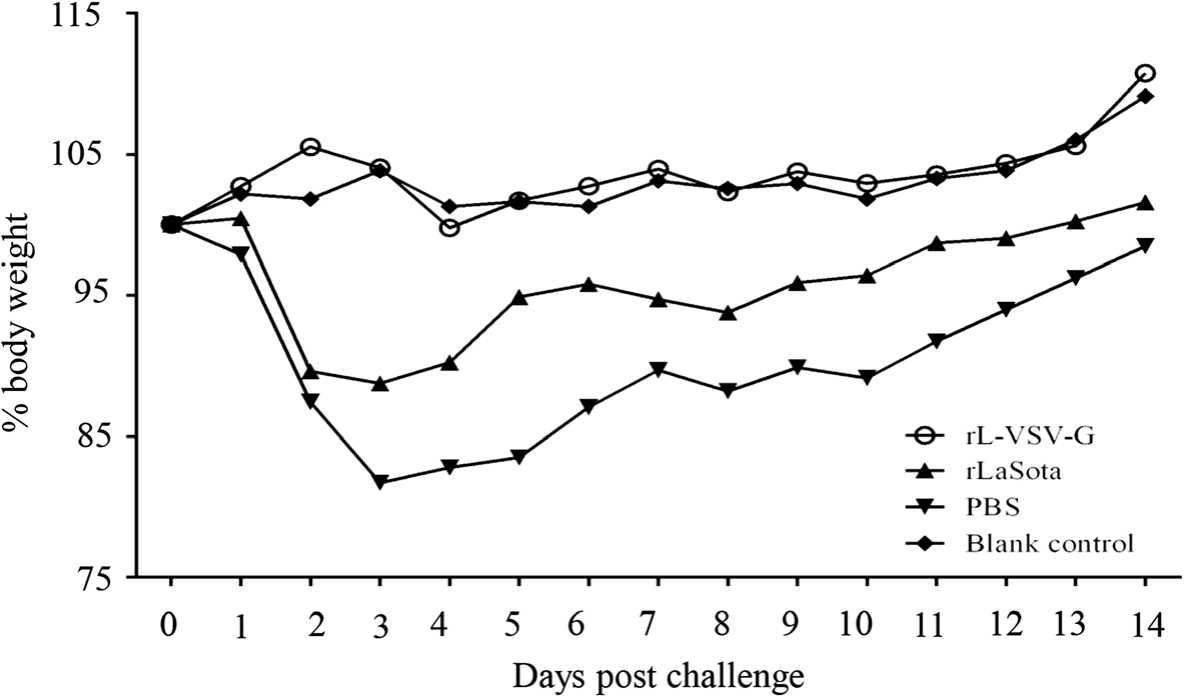
Changes in the weight of mice challenged with wild-type VSV Indiana strain. Two weeks after the boost immunization, mice were challenged with 1 × 10^7^ TCID_50_ of wt VSV Indiana strain by intranasal instillation. Mice were weighed daily for 14 days

**Fig. 5:**
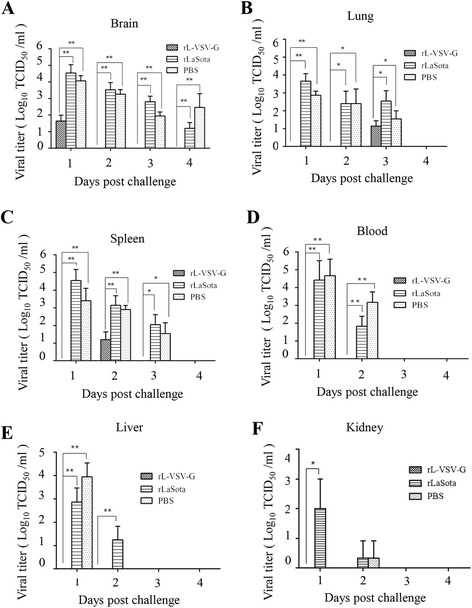
Viral loading in different organs of mice challenged with VSV Indiana strain. The mice were euthanized and viral loading was determined using inoculated BHK-21 cells from the (**a**) brain (**b**), lung (**c**), spleen (**d**), blood (**e**), liver, and (**f**) kidney in five days after challenge. Statistically significant differences were determined using the t test. *, *P* < 0.05

At second week after the boost immunization, the mice were challenged with wt VSV Indiana strain at 10^7^ TCID_50_ by intranasal instillation. After challenge, no deaths or typical symptoms of encephalitis were observed. Visible fluctuations in body weight were observed in rLaSota and PBS groups. There were no distinct changes in body weight after challenge in the rL-VSV-G group (Fig. 4). The mice were euthanized and the brains, lungs, livers, spleens, kidneys and blood were collected daily from first day one to day five after challenge. Viral titers in organs were tested in the BHK-21 cells. Results showed lower viral titers in the rL-VSV-G group than in other groups. wt VSV was isolated only from the brains, lungs and spleens of mice in the rL-VSV-G group, and viral titers were lower than that in other groups (Fig. 5). The viral load in rLaSota group was approximately equal to that of the PBS group, and wt VSV were detected from day one to day four after challenge.

### Assessment of protective efficacy of rL-VSV-G in suckling mice

**Fig. 6:**
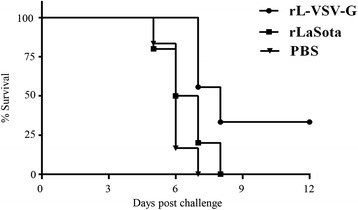
Passive protection assay of maternal antibody in suckling mice. Suckling mice were challenged with 10^3^ TCID_50_ of VSV Indiana strain by intranasal instillation on the 12th day after birth in a Biosafety Level-3 laboratory. The number of dead mice was recorded for 12 days after challenge

Before challenge, the neutralizing antibody titer against VSV for gravid mice was 1:512–1:1024 (data not shown) in rL-VSV-G group. The survival rate of suckling mice was 33 % in the rL-VSV-G group. In other groups it was 0 % (Fig. 6). In the rL-VSV-G group, some of the suckling mice died from seventh day post-challenge, while no mice died after the eighth day post-challenge. However, suckling mice in the PBS group died on the fifth day after challenge, and all died by seventh day. In the rLaSota group, the suckling mice died on the fifth day post-challenge and all died by eighth day. These results thus demonstrated that rL-VSV-G could provide good protection against VSV for adult mice and partial protection to suckling mice.

## Discussion

Vesicular stomatitis virus (VSV) causes severe losses to livestock industry and there are less available vaccines. The viral vector NDV has been used in many animals, such as African green monkey, rhesus monkey, pig, mouse, cattle, and chicken, as well as in humans [20, 22, 23, 26, 28–30, 34]. NDV vectored vaccines can be grown to high titers in embryonated chicken eggsand their safety and efficacy in human and animals are well documented [20, 22, 23, 26, 28–30, 34]. In this study, the recombinant NDV expressing VSV G (rL-VSV-G) was constructed. Our results showed rL-VSV-G was safe and immunogenic for mice. rL-VSV-G could induce high level of neutralizing antibody, which is likely due to the NDV vector has been reported to elicit strong innate immune responses in mammals [35, 36]. A previously study reported the cattle were protected when the SN titer was higher than 640 using vaccinia virus as vector [15]. Our results demonstrated rL-VSV-G could induce high level of neutralizing antibodies in mice (up to 760 after the second dose), thus we speculate the vaccine could confer protection to the immunized animals. Of note, immunization of susceptible animals such like pigs, cattle and horses are planned in the future studies.

Live-attenuated VSV vaccines were reported, such as neuro-virulence absence VSV with mutant M protein, could induce similar level of antibody to that of wt VSV [37]. A gene order re-arranged VSV which G gene was moved from its promoter-distal position to the first position in the genome [38]. The G protein expression was elevated in infected cells but not in virus particles. This re-arranged VSV induced higher neutralizing antibodies than that of wt VSV, but the viral titers was approximate10-fold lower than wt VSV. Compared with live-attenuated VSV, NDV-vectored VSV vaccine have several advantages: it has lower biosafety concerns; it is easy to culture and grow to high titers in chicken eggs; it does not require complicated cell culture equipment and are thermostable when lyophilized. Most importantly, it will not interfere with natural infection and vaccine immunization, thus can meet the requirement of DIVA vaccine.

In the challenge study, the mice did not appear to suffer any severe symptoms except for weight loss and convulsions in PBS group, however, these attenuated subclinical signs were similar to those of previous reports [16]. Nonetheless, the results regarding viral load in the tissues and body weight change were determined and supported the satisfactory the immune efficacy of rL-VSV-G.

To further examine the protective efficacy of recombinant rL-VSV-G, passive immunity experiment was performed. Obvious neurological symptoms including convulsions, shuddering, and death were observed in the PBS and rLaSota parental strain groups after challenge. However, the suckling mice, which had maternal antibodies against VSV showed attenuated clinical symptoms. In addition to this, there were also fewer suckling mice with neurological symptoms in the rL-VSV-G group than in other groups.

Additionally, differential roles of IgG subclasses in host defense have been studied most extensively for IgG1 and IgG2a, specifically addressing their ability to confer protection in models of viral and fungal infection [33, 39–41]. These studies have suggested that IgG2a is the most potent subclass in mediating protection [33, 42]. rL-VSV-G immunized mice tended to produce Th1 type immune response (IgG2a/IgG1 > 1), which probably contributed to good protective efficacy. In conclusion, all data demonstrated the recombinant virus rL-VSV-G was highly safe and immunogenic in mice. rL-VSV-G could induce high level of neutralizing antibodies and thus confer good protection against VSV challenge for mice. It appeared to be a promising candidate vaccine against VSV.

## Conclusions

Our results demostrated that rL-VSV-G could induce high level of neutralizing antibodies and confer good protection against VSV challenge for mice. It appeared to be a promising candidate vaccine against VSV.

## Methods

### Cells and viruses

Baby hamster kidney (BHK-21) cells were grown in Dulbecco’s modified Eagle’s medium essential medium (DMEM) containing 5 % fetal bovine serum (FBS). The NDV LaSota as vector virus was rescued from the genomic cDNA of the NDV LaSota vaccine strain (GenBank: AY845400.2) with additional help from MVA-T7 [22, 23]. Recombinant NDV strains, rLaSota and rLaSota expressing enhanced green fluorescence protein (EGFP) (rLaSota- EGFP) were grown and titrated in 10-day-old specific-pathogen-free (SPF) embryonated chicken eggs by allantoic cavity inoculation. Wild-type (wt) VSV (Indiana strain) and recombinant VSV expressing EGFP (rVSV-EGFP) were grown and titrated in BHK-21 cells.

### Rescue of recombinant virus

pBR322 containing NDV LaSota genomic cDNA have been reported previously [22]. The open reading frame (ORF) of G gene from VSV Indiana stain (Genbank Accession No. J02428.1) was produced by reverse transcription (RT)-PCR. Briefly, the VSV Indiana strain was grown in BHK-21 cells at a multiplicity of infection (MOI) of 0.01 TCID50 per cell over the course of 24 h (h), the supernatant was harvested and VSV genomic RNA was extracted with a Total RNA extraction Kit (OMEGA, Norcross, GA) according to the instructions provided with the kit. The G gene was amplified by RT-PCR using the following primer pair:

5′-GACTGTTTAAACTTAGAAAAAATACGGGTAGAAGTACTGCCACCatgaagtgccttttgtac-3′ and 5′-GACTGTTTAAACttactttccaagtcggttcatc-3′, in which the gene end and gene start sequences of NDV (underlined), the optimal Kozak sequence (italic), the Pme I restriction sites (bold) were introduced. The amplified G gene was sequenced and inserted into the LaSota genomic cDNA between P and M gene. The resultant plasmid (designated as pLa-VSV-G) was used for virus rescue as previously described [22]. The resultant recombinant virus was designated as rL-VSV-G.

### Immunofluorescence and western blot

The expression of G by the recombinant viruses was examined in BHK-21 cells by immunofluorescence and Western blot assays, using a mouse monoclonal antibody against VSV G protein (Sigma, U.S.) or chicken serum against NDV as previously described [22]. To confirm the expression of G in the recombinant virus, Western blot was performed using infected cell lysate. BHK-21 cells were infected with rLaSota or rL-VSV-G at a MOI = 1. After 24 h, the total cellular proteins were extracted with lysing buffer (1 % Nonidet P-40, 0.4 % deoxycholate, 50 mMTris-HCl [pH = 8], 62.5 mM EDTA) on ice for 5 min, and collected in 1.5 ml Eppendorf tubes, followed by centrifugation for 2 min at 15,000 × g. the supernatant was stored at −70 °C for Western blot analysis.

For confocal assay, BHK-21 cells were plated on cover slips in 35-mm-diameter dishes and infected with rLaSota or rL-VSV-G at a MOI = 0.01. The experimental procedure was performed. Briefly, at 48 h after infection, the cells were fixed and incubated with monoclonal antibodies against VSV G or mouse anti-NDV polyclonal IgG followed by FITC-conjugated goat anti-mouse antibody (Sigma, USA) and TRITC-conjugated rabbit anti-chicken antibody (Sigma, U.S.) [23]. Finally, cells were analyzed with a fluorescence microscope or confocal laser microscope. Images were acquired with a Zeiss Axioskop Fluorescent Microscope (Thornwood, NY, U.S.). It was equipped for epifluorescence with a Sensyscharge-coupled device camera (Photometrics,Tucson, AZ, U.S.) by using IPLab software (Scanalytics, Vienna, VA, U.S.).

### Pathogenicity in poultry and mice

To determine the pathogenicity of rL-VSV-G in poultry, the MDT, ICPI, and IVPI were determined in embryonated SPF chicken eggs or in SPF chickens according to the OIE Manual [43].

Forty-eight 4-week-old female Balb/c mice (Vital River, Beijing, China) were inoculated with 10^7^ TCID_50_ of rL-VSV-G or rLaSota by intranasal instillation (30 μl, 10^7^ TCID_50_) or intramuscular injection (100 μl, 10^7^ TCID_50_). Body weight was checked daily for 14 days. In five days after inoculation, mice tissues were collected and homogenized. Then viral tissue tropism was tested by indirect immunofluorescence (IFA) as described previously [22, 23].

### Evaluation of immune protective efficacy of the recombinant virus in mice

Eighty 4-week-old female mice (Balb/c) were randomly divided into four groups, and the groups were named rL-VSV-G, rLaSota, PBS, and naive control group, respectively. Twenty mice from the rL-VSV-G group were immunized with rL-VSV-G by intramuscular injection (100 μl, 10^7^ TCID_50_). Twenty mice were mock-infected with PBS (100 μl) and served as controls. An equal number of mice were inoculated intramuscularly with rLaSota (100 μl, 10^7^ TCID_50_). Booster immunization was performed at the third week after primary immunization. Blood samples were collected every week.

At second week after the boost immunization, the mice were challenged with wt VSV Indiana strain at 10^7^ TCID_50_ by intranasal instillation. This was performed in a Biosafety Level 3 laboratory. The body weight and symptoms of each mouse were monitored daily. The mice were euthanized and their organs were collected from first to fifth day after challenge and then stored in – 70 °C. The viral titer was calculated in BHK-21 using the Reed-Muench method [44].

### Suckling mice immunization and challenge assay

Fifteen 8-week-old female Balb/c mice were randomly divided into three groups. Five mice from each group were intramuscularly immunized with rL-VSV-G, rLaSota or PBS, according to an immunization program from above study. After the first week of initial inoculation, female mice from every group were allowed to share habitats with male mice for two weeks. Mouse pups were born during the fourth or fifth week after polyculture.

On the 12th day after birth, the suckling mice were challenged with wt VSV Indiana strain at 10^3^ TCID_50_ by intranasal instillation in a Biosafety Level 3 laboratory. The mice were monitored for 12 days for death or any clinical signs.

### ELISA

Enzyme-linked immunosorbent assay (ELISA) and neutralization assay were carried out to determine the humoral immune responses of immunized mice. For mouse serum, a previously reported procedure was used [26]: 96-well ELISA plates were coated overnight at 4 °C with inactivated purified VSV particles at a concentration of 4 μg/ml. The plates were then washed and blocked with 2 % BSA-PBST (PBS containing 0.05 % Tween-20 (v/v) and 2 % bovine serum albumin (BSA, wt/v)) at room temperature for 1 h. Serially diluted serum was added to the ELISA plate and incubated at room temperature for 1 h. Plates were washed three times with PBST, then a 1:4000 dilution of HRP-labeled goat anti-mouse IgG (Southern Biotech, Birmingham, AL, U.S.) was added and incubated for another 1 h at room temperature. The plates were washed thoroughly five times with PBST, and any remaining fluid was decanted completely from each plate. For visualization, 50 μl of 3,3′,5,5′-tetramethylbenzidine (TMB) liquid substrate (Sigma, U.S.) was added to each well for 5 min at room temperature; 50 μl of 0.2 M hydrochloric acid was added to stop the reaction. O.D. values were determined with a Model 680 microplate reader (Biorad) at 450 nm. A standard curve was generated by coating each ELISA plate with serial 2-fold dilutions of purified mouse IgG (Southern Biotech, Birmingham, AL, U.S.) at specific concentrations. A linear equation was produced based on the 2-fold decreased standard IgG concentration and their O.D. values, so the concentration of VSV G protein-specific antibodies in each sample could be calculated according to the linear equation using their O.D. values and expressed as the amount of antigen-specific IgG per ml of serum (ng/ml). As described above, the concentration of serum IgG2a and IgG1 were calculated and a similar process was performed.

### Serum neutralizing antibody titration

For the neutralization assay, sera were heat-inactivated at 56 °C for 30 min. Serial two-fold dilutions were mixed with equal volumes of virus were diluted to contain 500 TCID_50_/25 μl wt VSV-EGFP or 200 TCID_50_/25 μl rLasota-EGFP. The mixture was incubated for 60 min at 37 °C in 5 % CO_2_. Then, 50 μl of the serum-virus mixture was transferred to BHK-21 cell monolayers in 96-well plates and incubated for 1 h at 37 °C. The monolayers were then added to 100 μl of DMEM. After incubation for 48 h at 37 °C, IFA was administrated. Neutralization titers were expressed as the reciprocal of the highest dilution of serum that showed at least a 50 % reduction in the number of fluorescent cells relative to those of the negative control. This assay was performed as in a previous work [23].

### Ethics statements

The present study was carried out in strict accordance with the recommendations in the Guide for the Care and Use of Laboratory Animals of the Ministry of Science and Technology of the People’s Republic of China. The protocol was approved by the Animal Research Ethics Committee of Harbin Veterinary Research Institute, Chinese Academy of Agricultural Sciences.

## References

[1] Letchworth GJ, Rodriguez LL, Del Cbarrera J (1999). Vesicular stomatitis. Vet J.

[2] McCluskey BJ, Hurd HS, Mumford EL (1999). Review of the 1997 outbreak of vesicular stomatitis in the western United States. J Am Vet Med Assoc.

[3] Walton TE, Webb PA, Kramer WL, Smith GC, Davis T, Holbrook FR, Moore CG, Schiefer TJ, Jones RH, Janney GC (1982). Epizootic vesicular stomatitis in Colorado: epidemiologic and entomologic studies. Am J Trop Med Hyg.

[4] Mead DG, Ramberg FB, Besselsen DG, Mare CJ (2000). Transmission of vesicular stomatitis virus from infected to noninfected black flies co-feeding on nonviremic deer mice. Science.

[5] Mortimer EA (1978). Immunization against infectious disease. Science.

[6] Brink AJ, Richards GA (2015). Use of vaccines as a key antimicrobial stewardship strategy. S Afr Med J.

[7] de la Fuente J, Contreras M. Tick vaccines: current status and future directions. Expert Rev Vaccines. 2015;1–10. 10.1586/14760584.2015.1076339 26289976

[8] Marzi A, Feldmann H (2014). Ebola virus vaccines: an overview of current approaches. Expert Rev Vaccines.

[9] Chen H, Bu Z (2009). Development and application of avian influenza vaccines in China. Curr Top Microbiol Immunol.

[10] OIE Terrestrial Manual. Vesicular stomatitis chapter 2.01.19, 2015.

[11] Dubourget P, Lombard M, House JA, House C (2003). Protective immunity in cattle vaccinated with a commercial scale, inactivated, bivalent vesicular stomatitis vaccine. Vaccine.

[12] Roche S, Albertini AA, Lepault J, Bressanelli S, Gaudin Y (2008). Structures of vesicular stomatitis virus glycoprotein: membrane fusion revisited. Cell Mol Life Sci.

[13] Freer G, Burkhart C, Ciernik I, Bachmann MF, Hengartner H, Zinkernagel RM (1994). Vesicular stomatitis virus Indiana glycoprotein as a T-cell-dependent and -independent antigen. J Virol.

[14] Kelley JM, Emerson SU, Wagner RR (1972). The glycoprotein of vesicular stomatitis virus is the antigen that gives rise to and reacts with neutralizing antibody. J Virol.

[15] Mackett M, Yilma T, Rose JK, Moss B (1985). Vaccinia virus recombinants: expression of VSV genes and protective immunization of mice and cattle. Science.

[16] Cantlon JD, Gordy PW, Bowen RA (2000). Immune responses in mice, cattle and horses to a DNA vaccine for vesicular stomatitis. Vaccine.

[17] Nakaya T, Cros J, Park MS, Nakaya Y, Zheng H, Sagrera A, Villar E, Garcia-Sastre A, Palese P (2001). Recombinant Newcastle disease virus as a vaccine vector. J Virol.

[18] Bukreyev A, Collins PL (2008). Newcastle disease virus as a vaccine vector for humans. Curr Opin Mol Ther.

[19] Bukreyev A, Skiadopoulos MH, Murphy BR, Collins PL (2006). Nonsegmented negative-strand viruses as vaccine vectors. J Virol.

[20] DiNapoli JM, Nayak B, Yang L, Finneyfrock BW, Cook A, Andersen H, Torres-Velez F, Murphy BR, Samal SK, Collins PL, Bukreyev A (2010). Newcastle disease virus-vectored vaccines expressing the hemagglutinin or neuraminidase protein of H5N1 highly pathogenic avian influenza virus protect against virus challenge in monkeys. J Virol.

[21] DiNapoli JM, Yang L, Samal SK, Murphy BR, Collins PL, Bukreyev A (2010). Respiratory tract immunization of non-human primates with a Newcastle disease virus-vectored vaccine candidate against Ebola virus elicits a neutralizing antibody response. Vaccine.

[22] Ge J, Deng G, Wen Z, Tian G, Wang Y, Shi J, Wang X, Li Y, Hu S, Jiang Y (2007). Newcastle disease virus-based live attenuated vaccine completely protects chickens and mice from lethal challenge of homologous and heterologous H5N1 avian influenza viruses. J Virol.

[23] Ge J, Wang X, Tao L, Wen Z, Feng N, Yang S, Xia X, Yang C, Chen H, Bu Z (2011). Newcastle disease virus-vectored rabies vaccine is safe, highly immunogenic, and provides long-lasting protection in dogs and cats. J Virol.

[24] Khattar SK, Collins PL, Samal SK (2010). Immunization of cattle with recombinant Newcastle disease virus expressing bovine herpesvirus-1 (BHV-1) glycoprotein D induces mucosal and serum antibody responses and provides partial protection against BHV-1. Vaccine.

[25] Kortekaas J, de Boer SM, Kant J, Vloet RP, Antonis AF, Moormann RJ (2010). Rift Valley fever virus immunity provided by a paramyxovirus vaccine vector. Vaccine.

[26] Kong D, Wen Z, Su H, Ge J, Chen W, Wang X, Wu C, Yang C, Chen H, Bu Z (2012). Newcastle disease virus-vectored Nipah encephalitis vaccines induce B and T cell responses in mice and long-lasting neutralizing antibodies in pigs. Virology.

[27] Veits J, Wiesner D, Fuchs W, Hoffmann B, Granzow H, Starick E, Mundt E, Schirrmeier H, Mebatsion T, Mettenleiter TC, Romer-Oberdorfer A (2006). Newcastle disease virus expressing H5 hemagglutinin gene protects chickens against Newcastle disease and avian influenza. Proc Natl Acad Sci U S A.

[28] Goff PH, Krammer F, Hai R, Seibert CW, Margine I, Garcia-Sastre A, Palese P (2013). Induction of cross-reactive antibodies to novel H7N9 influenza virus by recombinant Newcastle disease virus expressing a North American lineage H7 subtype hemagglutinin. J Virol.

[29] Huang Z, Elankumaran S, Yunus AS, Samal SK (2004). A recombinant Newcastle disease virus (NDV) expressing VP2 protein of infectious bursal disease virus (IBDV) protects against NDV and IBDV. J Virol.

[30] Ge J, Wang X, Tian M, Gao Y, Wen Z, Yu G, Zhou W, Zu S, Bu Z (2015). Recombinant Newcastle disease viral vector expressing hemagglutinin or fusion of canine distemper virus is safe and immunogenic in minks. Vaccine.

[31] Ge J, Wang X, Tian M, Wen Z, Feng Q, Qi X, Gao H, Wang X, Bu Z (2014). Novel in-ovo chimeric recombinant Newcastle disease vaccine protects against both Newcastle disease and infectious bursal disease. Vaccine.

[32] Manual of Diagnostic Tests and Vaccines for Terrestrial Animals 2011 Office International des Epizooties, Paris. 2011.

[33] Coutelier JP, van der Logt JT, Heessen FW, Warnier G, Van Snick J (1987). IgG2a restriction of murine antibodies elicited by viral infections. J Exp Med.

[34] Buijs PR, Verhagen JH, van Eijck CH, van den Hoogen BG (2015). Oncolytic viruses: From bench to bedside with a focus on safety. Hum Vaccin Immunother.

[35] Nakaya Y, Nakaya T, Park MS, Cros J, Imanishi J, Palese P, Garcia-Sastre A (2004). Induction of cellular immune responses to simian immunodeficiency virus gag by two recombinant negative-strand RNA virus vectors. J Virol.

[36] Zamarin D, Martinez-Sobrido L, Kelly K, Mansour M, Sheng G, Vigil A, Garcia-Sastre A, Palese P, Fong Y (2009). Enhancement of oncolytic properties of recombinant newcastle disease virus through antagonism of cellular innate immune responses. Mol Ther.

[37] Ahmed M, Marino TR, Puckett S, Kock ND, Lyles DS (2008). Immune response in the absence of neurovirulence in mice infected with m protein mutant vesicular stomatitis virus. J Virol.

[38] Flanagan EB, Ball LA, Wertz GW (2000). Moving the glycoprotein gene of vesicular stomatitis virus to promoter-proximal positions accelerates and enhances the protective immune response. J Virol.

[39] Markine-Goriaynoff D, Coutelier JP (2002). Increased efficacy of the immunoglobulin G2a subclass in antibody-mediated protection against lactate dehydrogenase-elevating virus-induced polioencephalomyelitis revealed with switch mutants. J Virol.

[40] Markine-Goriaynoff D, van der Logt JT, Truyens C, Nguyen TD, Heessen FW, Bigaignon G, Carlier Y, Coutelier JP (2000). IFN-gamma-independent IgG2a production in mice infected with viruses and parasites. Int Immunol.

[41] Taborda CP, Rivera J, Zaragoza O, Casadevall A (2003). More is not necessarily better: prozone-like effects in passive immunization with IgG. J Immunol.

[42] Nimmerjahn F, Bruhns P, Horiuchi K, Ravetch JV (2005). FcgammaRIV: a novel FcR with distinct IgG subclass specificity. Immunity.

[43] OIE manual. Manual of Diagnostic Tests and Vaccines for Terrestrial Animals 2011. Office International des Epizooties: Paris; 2011.

[44] Reed LJ, Muench H (1938). A simple method of estimating fifty per cent endpoints. Am J Epidemiol.

